# A Rare Case of Olmesartan-Associated Enteropathy Successfully Managed With Steroid Taper

**DOI:** 10.7759/cureus.41604

**Published:** 2023-07-09

**Authors:** Angela Xue, Mark R Fowler, Jan Silverman, Emily Sturkie, Evan Raff

**Affiliations:** 1 Medicine, University of North Carolina at Chapel Hill School of Medicine, Chapel Hill, USA; 2 Pathology and Laboratory Medicine, University of North Carolina at Chapel Hill School of Medicine, Chapel Hill, USA

**Keywords:** olmesartan, olmesartan-induced enteropathy, intestinal diseases, diarrhea, olmesartan medoxomil, angiotensin receptor antagonists

## Abstract

Olmesartan is a commonly used antihypertensive medication belonging to the class of angiotensin II receptor blockers. Though generally well-tolerated, olmesartan can rarely cause olmesartan-associated enteropathy (OAE) with non-bloody diarrhea, weight loss, abdominal pain, and vomiting. Patients may develop enteropathy months to years after drug initiation. In severe cases, patients may develop complications that require hospitalization. Diagnosis is often delayed due to unfamiliarity of OAE, nonspecific presenting symptoms, and normal-appearing gross endoscopic findings. Esophagogastroduodenoscopy (EGD) with biopsy is essential to the diagnosis, showing sprue-like enteropathy with intestinal villous atrophy and mucosal inflammation.

This report describes a case of a 70-year-old man who presented with three months of profuse watery diarrhea and 40-pound unintentional weight loss. After an extensive workup, including EGD with duodenal biopsies, the patient was diagnosed with OAE. The biopsies showed findings consistent with acute and chronic duodenitis, mucosal desquamation and ulceration, blunting of villi, and a sprue-like pattern with neutrophils. Celiac serologies and anti-enterocyte antibodies were negative, further supporting the diagnosis of OAE. Complete resolution of symptoms was achieved by discontinuing olmesartan and administering a steroid taper.

Considering the frequent use of olmesartan, the increasing occurrence of OAE, and the wide range of associated symptoms, it is crucial for providers to recognize OAE and consider early discontinuation of olmesartan. This approach can help prevent further intestinal damage, protracted symptoms, unnecessary diagnostic tests, and financial burdens on both patients and the healthcare system.

## Introduction

Systemic arterial hypertension (HTN) is a significant yet modifiable risk factor for various diseases, such as stroke, coronary artery disease (CAD), and chronic kidney disease (CKD). The use of antihypertensive medications has been shown to substantially reduce all-cause mortality and the risk of CAD among patients with baseline systolic blood pressure greater than 140 mmHg.

As one of the first-line antihypertensives, angiotensin receptor blockers (ARBs) selectively inhibit angiotensin II receptors through competitive inhibition, subsequently preventing angiotensin II-induced vasoconstriction and aldosterone release [1]. Olmesartan is a highly effective and largely safe ARB for patients with HTN [2]. In 2020, 1,163,607 patients in the United States used olmesartan, making it the 139th most prescribed medication [3]. Although generally well-tolerated, olmesartan has been rarely reported to cause olmesartan-associated enteropathy (OAE), imparting a sprue-like pattern on intestinal biopsy [4]. OAE presents as severe chronic diarrhea months to years after initiation of olmesartan [5].

The nonspecific symptoms of OAE, such as diarrhea, weight loss, and abdominal pain, frequently lead to extensive work-up and healthcare expenditure before reaching the correct diagnosis. Here, we present a 70-year-old man with profuse diarrhea and subsequent 40-pound weight loss who was later diagnosed with OAE through extensive medical testing. It is extremely crucial for clinicians to recognize OAE and discontinue olmesartan as soon as possible to prevent over-testing and prolonged patient suffering.

## Case presentation

A 70-year-old man presented to the emergency department (ED) with a three-month history of profuse watery diarrhea and 40-pound weight loss. His medical history included HTN, CKD, and idiopathic thrombocytopenic purpura (ITP). Two months prior, he was seen at an outside facility where he underwent extensive non-diagnostic work-up of his symptoms with lab testing, imaging, esophagogastroduodenoscopy (EGD), and colonoscopy. At that time, his serum pancreatic elastase-1 level was low, which prompted a possible diagnosis of pancreatic insufficiency. However, the initiation of supplemental pancreatic enzymes provided minimal improvement in his symptoms.

Upon evaluation, he was afebrile with a temperature of 36.8°C and tachycardic with a heart rate of 103 bpm. A physical exam revealed no abdominal tenderness, rebound, guarding, or hepatosplenomegaly. Medications included lipase-protease-amylase, dicyclomine, olmesartan, amlodipine, and loratadine; the former two were started the preceding two months with the latter being chronic medications on which he had been for at least one year prior to symptom onset.

During hospitalization, the patient consistently had substantial daily stool output, with the maximum reaching 6.25 L over 24 hours. Based on the large volume of stool output, secretory diarrhea was initially suspected, for which empiric octreotide was initiated. Although this briefly improved his symptoms, the patient quickly relapsed. Meanwhile, an extensive laboratory work-up was pursued.

Stool electrolytes were consistent with osmotic diarrhea with an osmotic gap of 112 mOsm/kg. Multiple digestive enzymes and markers were outside of the normal reference range, including fecal calprotectin (84.3 mcg/g; normal <= 50 mcg/g), serum chromogranin A (527 ng/mL; normal < 93 ng/mL), gastrin (169 pg/mL; normal < 100 pg/mL), and pancreatic elastase (69 mcg/g; normal > 200 mcg/g). The patient had positive serum *Strongyloides* antibody, but normal fecal alpha-1-anti-trypsin. Negative stool studies included *Helicobacter pylori* antigen, cathartic screen (stool magnesium and phosphorous content), comprehensive ova and parasite panel, and *Clostridium difficile* assay. Various other serum tests were all within normal limits, including 5-hydroxyindoleacetic acid, thyroid-stimulating hormone, free T4, vitamins B3/B12/D, tryptase, tissue transglutaminase (TTG) IgA, vasoactive intestinal peptide, immunoglobulins (IgG, IgM, IgE, and IgA), human immunodeficiency virus antigen/antibody, glucagon, C-reactive protein, free light chains, somatostatin, viral hepatitis A/B/C serologies, and cytomegalovirus IgM/IgG. Magnetic resonance imaging (MRI) of the abdomen with/without contrast and contrasted computed tomography (CT) and CT enterography of the abdomen and pelvis demonstrated no significant pathology.

**Figure 1:**
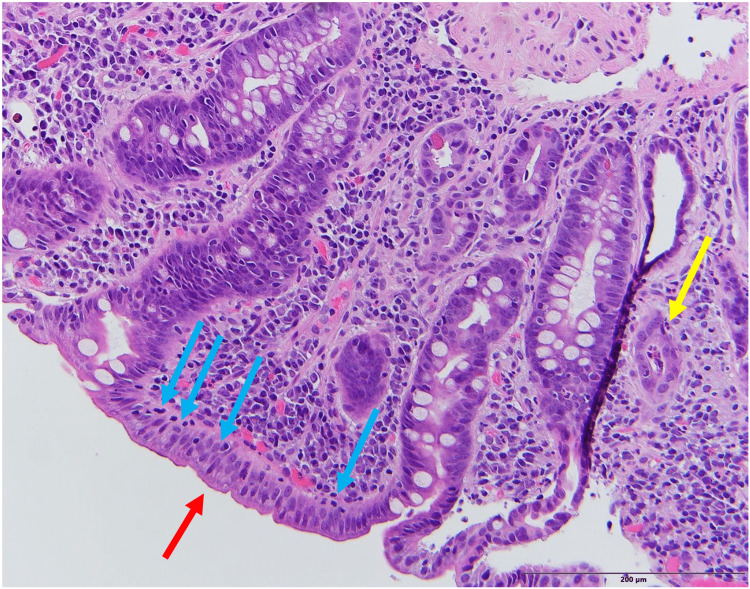
Low power (H&E x 20) duodenal biopsy showing complete villous atrophy (red arrow) and moderate chronic active duodenitis (modified Marsh-Oberhuber type 3c). There are moderately increased intraepithelial luminal lymphocytes (blue arrows) and one crypt abscess (yellow arrow). H&E: hematoxylin and eosin.

**Figure 2:**
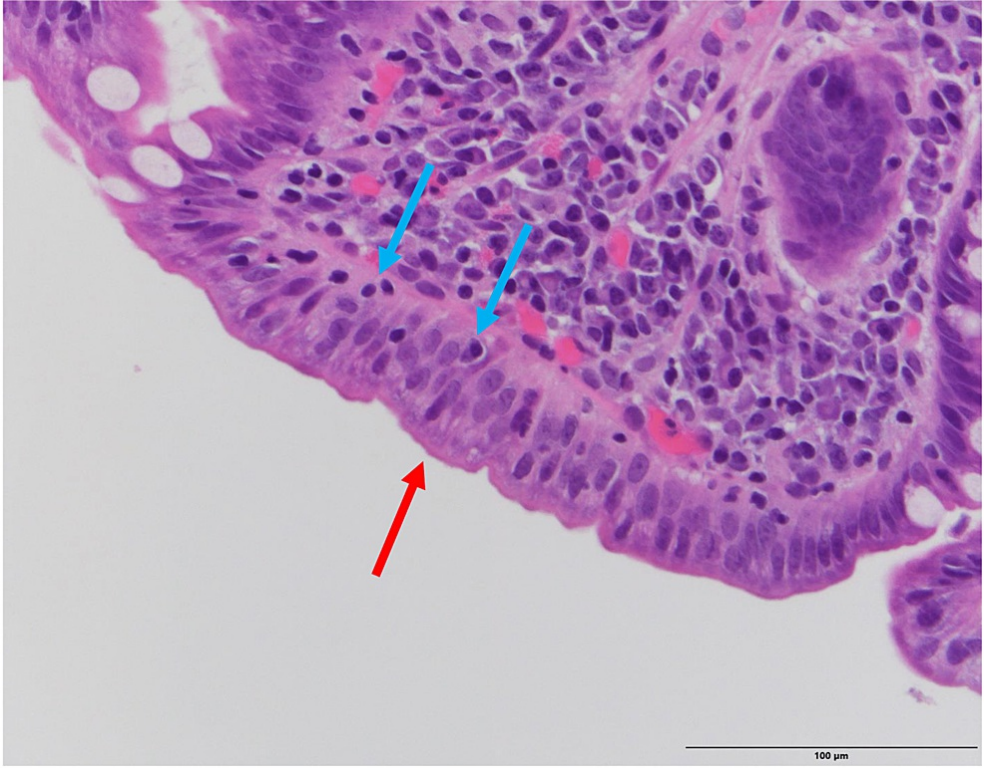
High power (H&E x 40) duodenal biopsy showing complete villous atrophy (red arrow) and moderate chronic active duodenitis. There are moderately increased intraepithelial luminal mucosal lymphocytes (blue arrows). H&E: hematoxylin and eosin.

EGD and colonoscopy revealed grossly normal upper and lower gastrointestinal tracts, respectively. Random duodenal biopsies demonstrated moderate acute and chronic duodenitis with mucosal desquamation and ulceration, few intact villi with blunting, increased intraluminal lymphocytes, and a few neutrophils imparting a sprue-like pattern (Figures 1, 2). Random biopsies of the stomach showed an unremarkable gastric mucosa with no evidence of *Helicobacter pylori* on hematoxylin and eosin (H&E) examination. Random colonic biopsies showed normal colonic mucosa.

**Figure 3:**
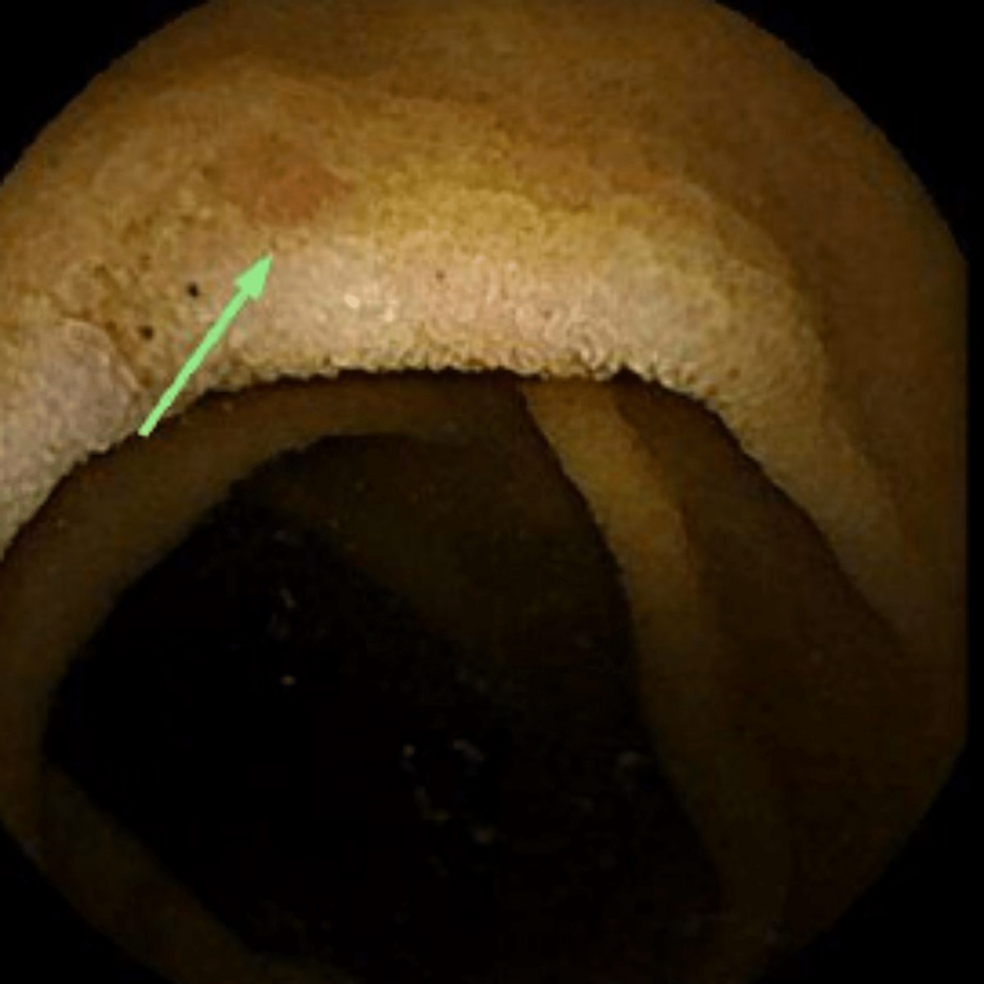
VCE image showing the duodenal bulb with diffuse granularity, abnormal mucosa, and area of erosion (green arrow). VCE: video capsule endoscopy.

**Figure 4:**
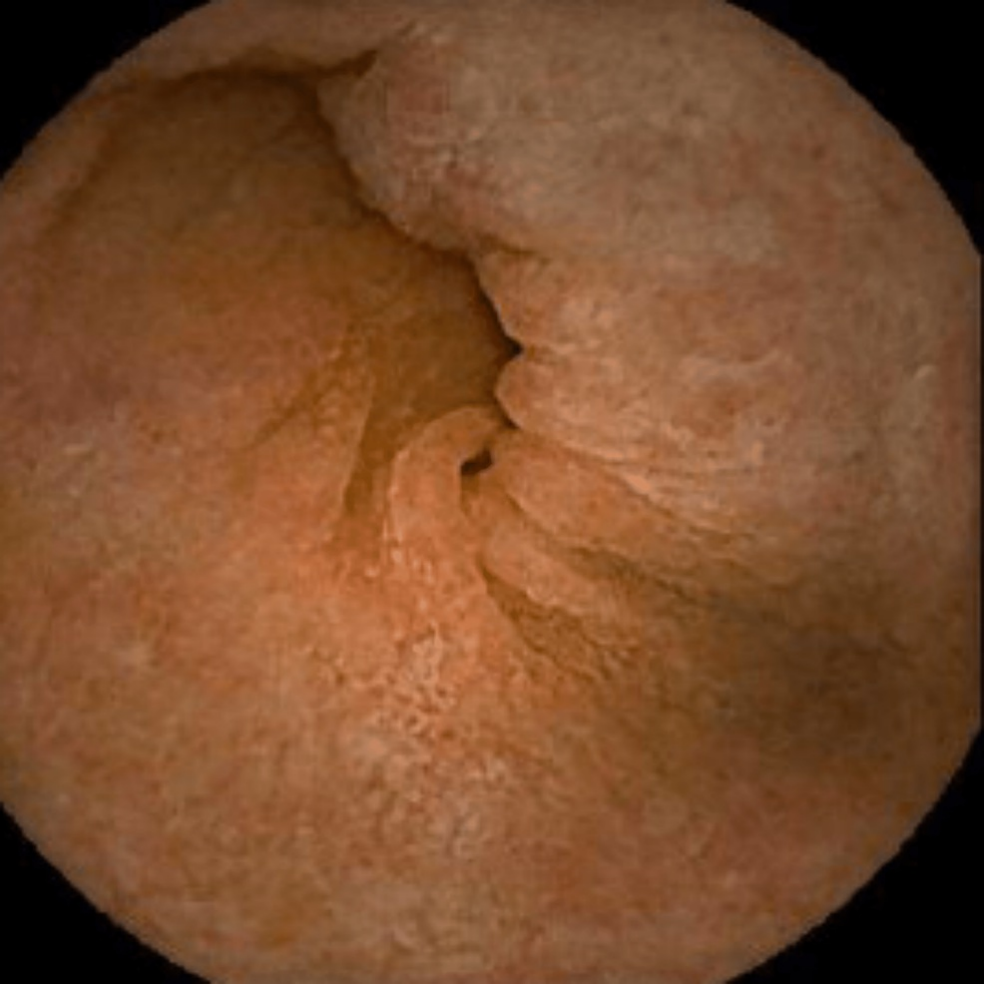
VCE image showing the absence of villi in the proximal jejunum. VCE: video capsule endoscopy.

**Figure 5:**
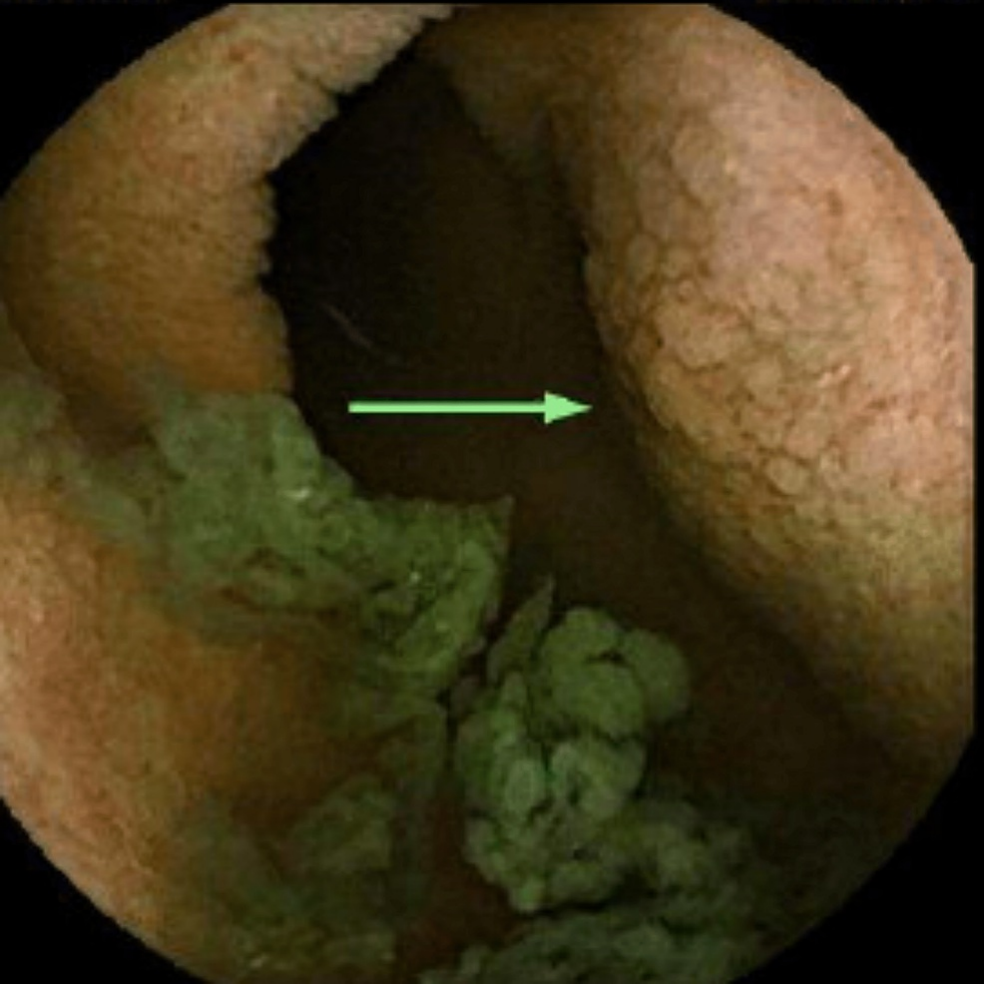
VCE image showing abnormal mucosa with granularity and no clear villi (green arrow) in the proximal ileum. VCE: video capsule endoscopy.

**Figure 6:**
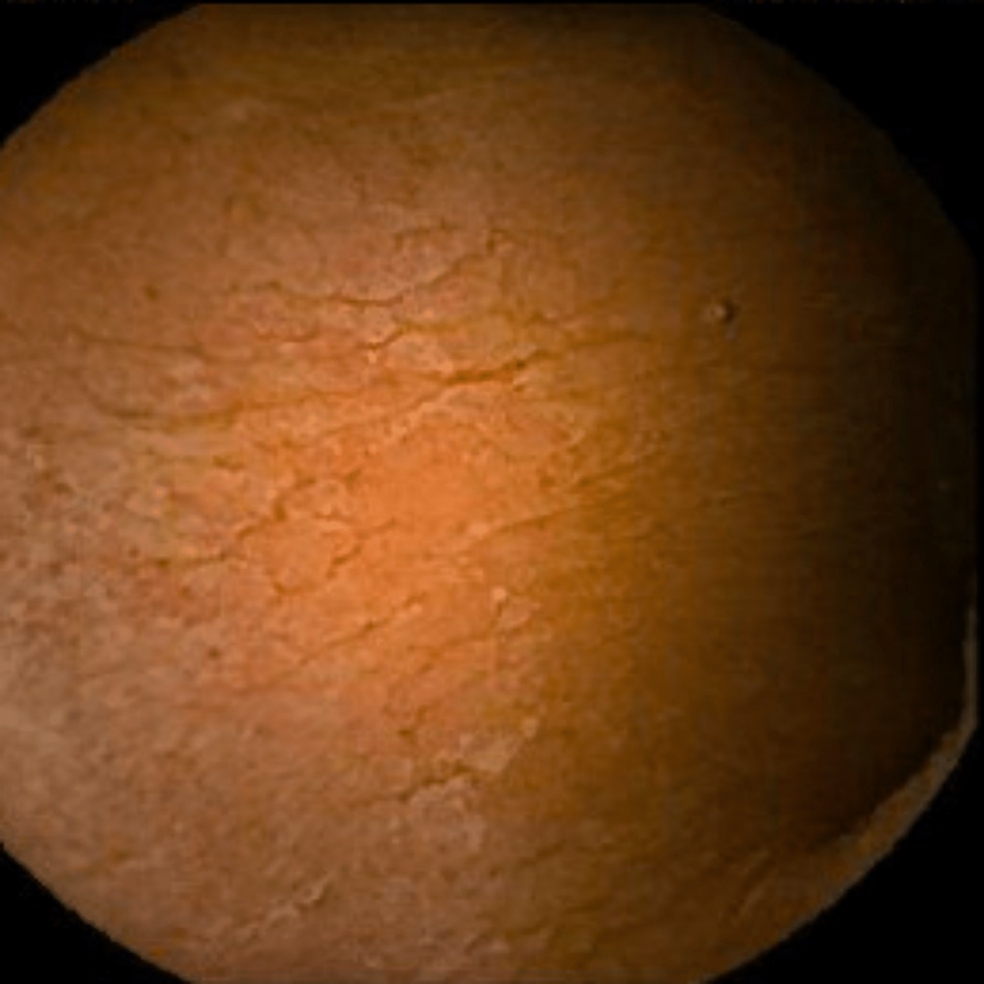
VCE image showing granularity and denuded mucosa with fissuring across a diffuse area of the mid-ileum. VCE: video capsule endoscopy.

Since the diagnosis remained in question and no gross findings were noted on EGD and colonoscopy, the gastrointestinal consult provider performed a video capsule endoscopy (VCE). The VCE showed granularity with apparent villous flattening throughout the small bowel as well as mucosal denudation and fissuring in the distal small bowel suggestive of severe enteropathy (Figures 3-6).

The differential for sprue-like enteropathy includes OAE, celiac disease, and autoimmune enteropathy. Considering the patient’s use of olmesartan, severe diarrhea and associated weight loss, and negative celiac serology and anti-enterocyte antibody, the diagnosis of OAE was established. In addition to olmesartan cessation, methylprednisolone followed by a prednisone taper resulted in the patient’s complete symptom resolution. He was subsequently discharged and gained four pounds at his follow-up outpatient visit two weeks later. His symptoms have not recurred since the discontinuation of olmesartan.

## Discussion

OAE is thought to be a rare yet serious condition that can be easily overlooked, in part due to its nonspecific symptoms. Rubio-Tapia et al. were the first to identify the association between olmesartan use and sprue-like enteropathy [6]. They identified 22 patients at the Mayo Clinic in Rochester, Minnesota who had unexplained chronic diarrhea and enteropathy and experienced symptom and histologic improvement upon cessation of olmesartan [6]. Since then, there have been a few additional case reports describing similar presentations of OAE [7-10]. Although the exact pathophysiology of OAE is unknown, a systematic review including 82 case reports and case series found that 71.4% of olmesartan users had human leucocyte antigen (HLA) haplotypes (HLA-DQ2 and HLA-DQ8) [11]. A biochemical study further revealed that OAE leads to interleukin (IL)-15 overexpression by epithelial cells and disruption of tight junction protein ZO-1 [12]. Additionally, olmesartan has been found to have a stronger affinity for angiotensin type I (AT1) receptors than angiotensin type II (AT2) receptors [11,13]. In the presence of olmesartan, the increased proportion of available AT2 receptors leads to amplified binding of angiotensin, which stimulates apoptosis of intestinal epithelial cells and the subsequent development of villous atrophy [11,13].

OAE frequently presents with nonspecific gastrointestinal symptoms such as non-bloody diarrhea, weight loss, crampy abdominal pain, nausea, and vomiting [10]. Cutaneous involvement, including acquired bullous epidermolysis, has also been reported [8]. In severe cases, patients may develop acute kidney failure requiring renal replacement therapy [14]. OAE can develop months to years after the initiation of olmesartan [15]. The diagnosis is commonly delayed due to nonspecific presenting symptoms, late onset of symptoms following the start of olmesartan, and unfamiliarity with the condition. The wide spectrum of nonspecific gastrointestinal symptoms in OAE frequently leads to extensive workups for other more common disease entities. Intestinal biopsy showing sprue-like enteropathy with villous atrophy and mucosal inflammation is essential to the diagnosis of OAE [15,16]. Unfortunately, OAE can have normal-appearing endoscopic findings on gross examination, prompting no pursuit of biopsy and continued delay in diagnosis. The pathologic findings in OAE are like those of celiac disease and autoimmune enteropathy, thus ruling out these conditions by history and anti-TTG/IgA and anti-enterocyte antibody testing, respectively, is of paramount importance. In this case, it was only after an extensive work-up that the diagnosis of OAE was established.

Treatment of OAE requires olmesartan cessation with the addition of oral or intravenous (IV) steroids to help mitigate symptoms in severe cases. Once olmesartan is discontinued, clinical improvement typically occurs within a few days to one week [15-17]. In this case, discontinuation of olmesartan and initiation of steroid therapy resulted in the complete resolution of the patient’s symptoms without recurrence. Although olmesartan is the most common ARB to cause sprue-like enteropathy, multiple case reports have described other ARBs capable of causing a similar complication [11,17,18]. Therefore, in any patients on ARB medications presenting with nonspecific gastrointestinal symptoms, providers should consider discontinuation of the ARB regardless of the specific type.

Interestingly, conflicting evidence has been reported on whether olmesartan use is associated with an increased risk of enteropathy. A retrospective cohort study utilizing Korean National Health Insurance Service data (n = 108,559) found that the incidence rate ratio of enteropathy between olmesartan and angiotensin-converting enzyme (ACE) inhibitors (0.33, 95% CI: 0.10-1.09) was not statistically significant [19]. However, Kang and colleagues recently identified the first case of OAE in Korea, indicating that OAE may be extremely rare in the Korean population but does exist [14]. Additionally, Basson et al. conducted a nationwide cohort study using the French National Health Insurance claim database and found that the adjusted incidence rate ratio for hospitalization due to intestinal malabsorption secondary to olmesartan compared to intestinal malabsorption patients taking ACE inhibitors is 2.49 (95% CI: 1.73-3.57) [20]. More research is warranted on both the prevalence and incidence of OAE in the global community and the geographical and ethnic differences of this condition.

## Conclusions

Healthcare providers should be familiar with OAE as a rare cause of chronic diarrhea, weight loss, and malnutrition. A lack of familiarity with OAE, its non-specific symptoms, and the variable delay in symptom onset after olmesartan initiation make the diagnosis challenging. These same factors frequently contribute to extensive diagnostic testing that places patients at unnecessary risk and imparts significant financial and social burdens on both patients and the healthcare system. Given these considerations and the high frequency of olmesartan usage, it is crucial for providers to recognize this disease entity and consider early discontinuation of olmesartan.
